# Subcellular proteomics—where cell biology meets protein chemistry

**DOI:** 10.3389/fpls.2014.00055

**Published:** 2014-02-26

**Authors:** A. Harvey Millar, Nicolas L. Taylor

**Affiliations:** ARC Centre of Excellence in Plant Energy Biology, Centre for Comparative Analysis of Biomolecular Networks, The University of Western AustraliaPerth, WA, Australia

**Keywords:** sub-cellular proteomics, mass spectrometry, organelles, model plants, crop plants

The development of compartments in eukaryotic cells and the distribution of nuclear-encoded proteins underlies the expansion of plant genomes, the proliferation of multigene families and the specialization of cellular functions. The exploration of the proteome of the cell in terms of the collection of its subcompartments is therefore both a practical approach and also a function led necessity that recognizes that proper interpretation of proteomic data requires information about compartmentation of protein machinery.

Subcellular proteomics decreases the complexity of proteome discovery. With the typical compartment representing 500–4000 proteins, its analysis by gel based and MS based systems approach the resolution of the analytical techniques. In contrast, whole cell proteomes of 12,000–40,000 proteins extend well beyond the ability of proteomic tools to resolve them, leaving whole cell proteome studies being “tip of the iceberg” activities. Current shotgun studies can identify ~500–3000 proteins with 2–20 h of MS time, making organelle proteomes and their quantitative comparisons within the reach of many research laboratories that either perform their own MS or use MS services.

Subcellular proteomics stands on the shoulders of decades of biochemical research that has developed methods for isolation of subcellular compartments. Extensive laboratory work involving the tinkering with density, size, and charge separation techniques has enabled incremental limitation of contamination in isolation methods from a range of subcellular structures. However, in depth MS studies over the last decade have also revealed that typical 90–95% enrichment still leaves much room for contaminants in preparations (Eubel et al., 2007; Huang et al., 2009; Ito et al., 2014). Studies from relatively abundant, or easily isolated homogenous compartments dominate the literature. In this class of structures are plastids, mitochondria, peroxisomes, and nuclei. Currently over 8000 proteins have been experimentally identified in these organelles in Arabidopsis (Tanz et al., 2013). Many fewer studies have attempted to untangle the intracellular membrane systems of ER, golgi, and PM. Separate techniques for these are complex, lack high levels of enrichment, and the protein populations of these structures are often transitory and differ between tissue types. Currently over 6000 proteins have been experimentally identified in these membranes in Arabidopsis (Tanz et al., 2013). All these structures bathe in the cytosol of the cell that itself contains a large and complex proteome. Isolation of pure cytosol without breaking organelles is extremely challenging and thus cytosolic proteomes are best defined through subtractive analysis of soluble proteomes against enriched organelle datasets. Quantitative comparisons of fractions collected during the subcompartment enrichment process, or across gradient separation of organelles, are key tools to differentiate the low level protein component from the small fraction of a contaminating protein from another location in the cell. Bringing together subcellular proteomics studies in aggregation databases has been very revealing to confirm location of proteins for which there are multiple conflicting claims in the literature (Tan et al., 2012; Tanz et al., 2013).

Analysis of multiple subcellular proteomes from the same tissues has begun to show the way in which multigene families have dispersed particular protein classes across subcellular boundaries to maintain translational, metabolic, signaling, and degradative machinery through the cell. Subcellular proteomes and targeted metabolic engineering are also showing how steps in metabolic pathways have been, and can be, redistributed in plants (compared to animals) to enable unique chemistries and accumulation of end-products in plants.

This special research topic aimed to bring together knowledge across sub cellular components and plant species to provide a basis for accelerated research in plant subcellular protein research. We have brought together a wide array of 26 publications including original research articles, reviews, and mini-reviews. They are focused on the model plants Arabidopsis (Parsons et al., 2012; Albenne et al., 2013; Bussell et al., 2013; Carroll, 2013; Lee et al., 2013a; Peters et al., 2013; Simm et al., 2013; Yadeta et al., 2013; Ito et al., 2014), rice (Huang et al., 2013; Komatsu and Yanagawa, 2013) and medicago (Kiirika et al., 2013; Lee et al., 2013b; Simm et al., 2013) as well as crop plants wheat, barley, maize, and tomato (Casati, 2012; Komatsu and Yanagawa, 2013; Petersen et al., 2013; Ruiz-May and Rose, 2013; Zhang et al., 2013). They include studies of the easily isolated subcellular proteomes of the chloroplast, mitochondria, peroxisome, and nuclei (Casati, 2012; Repetto et al., 2012; Bussell et al., 2013; Havelund et al., 2013; Huang et al., 2013; Lee et al., 2013a; Narula et al., 2013; Peters et al., 2013; Petersen et al., 2013; Simm et al., 2013), as well as less easily isolated golgi, plasma membrane, cytosolic ribosome, and cell wall proteomes(Parsons et al., 2012; Carroll, 2013; Takahashi et al., 2013; Yadeta et al., 2013; Zhang et al., 2013). Articles have also begun to investigate sub-organellar proteomes including the subcompartments of chloroplast (Simm et al., 2013) and mitochondria (Peters et al., 2013), plasma membrane microdomains (Takahashi et al., 2013), and cell wall plasmodesmata (Salmon and Bayer, 2012). In addition to cataloguing these proteomes, researchers are beginning to investigate the posttranslational modifications present on proteins in these locations (Havelund et al., 2013).

## References

[B1] Albenne C. Canut H. Jamet E. (2013). Plant cell wall proteomics: the leadership of *Arabidopsis thaliana*. Front. Plant Sci. 4:111 10.3389/fpls.2013.00111 23641247 PMC3640192

[B2] Bussell J. D. Behrens C. Ecke W. Eubel H. (2013). Arabidopsis peroxisome proteomics. Front. Plant Sci. 4:101 10.3389/fpls.2013.00101 PMC3633942 23630535

[B3] Carroll A. J. (2013). The Arabidopsis cytosolic ribosomal proteome: from form to function. Front. Plant Sci. 4:32 10.3389/fpls.2013.00032 23459595 PMC3585428

[B4] Casati P. (2012). Recent advances in maize nuclear proteomic studies reveal histone modifications. Front. Plant Sci. 3:278 10.3389/fpls.2012.00278 23248634 PMC3520088

[B5] Eubel H. Lee C. P. Kuo J. Meyer E. H. Taylor N. L. Millar A. H. (2007). Free-flow electrophoresis for purification of plant mitochondria by surface charge. Plant J. 52, 583–594 10.1111/j.1365-313X.2007.03253.x 17727614

[B6] Havelund J. F. Thelen J. J. Moller I. M. (2013). Biochemistry, proteomics, and phosphoproteomics of plant mitochondria from non-photosynthetic cells. Front. Plant Sci. 4:51 10.3389/fpls.2013.00051 23494127 PMC3595712

[B7] Huang S. Shingaki-Wells R. N. Taylor N. L. Millar A. H. (2013). The rice mitochondria proteome and its response during development and to the environment. Front. Plant Sci. 4:16 10.3389/fpls.2013.00016 PMC3566409 23403394

[B8] Huang S. Taylor N. L. Narsai R. Eubel H. Whelan J. Millar A. H. (2009). Experimental analysis of the rice mitochondrial proteome, its biogenesis, and heterogeneity. Plant Physiol. 149, 719–734 10.1104/pp.108.131300 19010998 PMC2633852

[B9] Ito J. Parsons H. T. Heazlewood J. L. (2014). The Arabidopsis cytosolic proteome: the metabolic heart of the cell. Front. Plant Sci. 5:21 10.3389/fpls.2014.00021 24550929 PMC3914213

[B10] Kiirika L. M. Behrens C. Braun H. P. Colditz F. (2013). The Mitochondrial complexome of *Medicago truncatula*. Front. Plant Sci. 4:84 10.3389/fpls.2013.00084 23596449 PMC3625726

[B11] Komatsu S. Yanagawa Y. (2013). Cell wall proteomics of crops. Front. Plant Sci. 4:17 10.3389/fpls.2013.00017 23403621 PMC3566523

[B12] Lee C. P. Taylor N. L. Millar A. H. (2013a). Recent advances in the composition and heterogeneity of the Arabidopsis mitochondrial proteome. Front. Plant Sci. 4:4 10.3389/fpls.2013.00004 23355843 PMC3554846

[B13] Lee J. Lei Z. Watson B. S. Sumner L. W. (2013b). Sub-cellular proteomics of *Medicago truncatula*. Front. Plant Sci. 4:112 10.3389/fpls.2013.00112 23641248 PMC3639374

[B14] Narula K. Datta A. Chakraborty N. Chakraborty S. (2013). Comparative analyses of nuclear proteome: extending its function. Front. Plant Sci. 4:100 10.3389/fpls.2013.00100 23637696 PMC3636469

[B15] Parsons H. T. Christiansen K. Knierim B. Carroll A. Ito J. Batth T. S. (2012). Isolation and proteomic characterization of the Arabidopsis Golgi defines functional and novel components involved in plant cell wall biosynthesis. Plant Physiol. 159, 12–26 10.1104/pp.111.193151 22430844 PMC3375956

[B16] Peters K. Belt K. Braun H. P. (2013). 3D Gel Map of Arabidopsis Complex I. Front. Plant Sci. 4l:153 10.3389/fpls.2013.00153 23761796 PMC3671202

[B17] Petersen J. Rogowska-Wrzesinska A. Jensen O. N. (2013). Functional proteomics of barley and barley chloroplasts - strategies, methods and perspectives. Front. Plant Sci. 4:52 10.3389/fpls.2013.00052 23515231 PMC3600771

[B18] Repetto O. Rogniaux H. Larre C. Thompson R. Gallardo K. (2012). The seed nuclear proteome. Front. Plant Sci. 3:289 10.3389/fpls.2012.00289 23267364 PMC3526781

[B19] Ruiz-May E. Rose J. K. (2013). Progress toward the tomato fruit cell wall proteome. Front. Plant Sci. 4:159 10.3389/fpls.2013.00159 PMC3665905 23755055

[B20] Salmon M. S. Bayer E. M. (2012). Dissecting plasmodesmata molecular composition by mass spectrometry-based proteomics. Front. Plant Sci. 3:307 10.3389/fpls.2012.00307 PMC3542633 23335932

[B21] Simm S. Papasotiriou D. G. Ibrahim M. Leisegang M. S. Muller B. Schorge T. (2013). Defining the core proteome of the chloroplast envelope membranes. Front. Plant Sci. 4:11 10.3389/fpls.2013.00011 23390424 PMC3565376

[B22] Takahashi D. Kawamura Y. Uemura M. (2013). Detergent-resistant plasma membrane proteome to elucidate microdomain functions in plant cells. Front. Plant Sci. 4:27 10.3389/fpls.2013.00027 PMC3579295 23440896

[B23] Tan Y. F. Millar A. H. Taylor N. L. (2012). Components of mitochondrial oxidative phosphorylation vary in abundance following exposure to cold and chemical stresses. J. Proteome Res. 11, 3860–3879 10.1021/pr3003535 22574745

[B24] Tanz S. K. Castleden I. Hooper C. M. Vacher M. Small I. Millar H. A. (2013). SUBA3: a database for integrating experimentation and prediction to define the SUBcellular location of proteins in Arabidopsis. Nucleic Acids Res. 41, D1185–D1191 10.1093/nar/gks1151 23180787 PMC3531127

[B25] Yadeta K. A. Elmore J. M. Coaker G. (2013). Advancements in the analysis of the Arabidopsis plasma membrane proteome. Front. Plant Sci. 4:86 10.3389/fpls.2013.00086 23596451 PMC3622881

[B26] Zhang Z. Voothuluru P. Yamaguchi M. Sharp R. E. Peck S. C. (2013). Developmental distribution of the plasma membrane-enriched proteome in the maize primary root growth zone. Front. Plant Sci. 4:33 10.3389/fpls.2013.00033 23508561 PMC3589600

